# Transcriptional Regulation of the IGF Signaling Pathway by Amino Acids and Insulin-Like Growth Factors during Myogenesis in Atlantic Salmon

**DOI:** 10.1371/journal.pone.0011100

**Published:** 2010-06-14

**Authors:** Neil I. Bower, Ian A. Johnston

**Affiliations:** Scottish Oceans Institute, School of Biology, University of St Andrews, St Andrews, United Kingdom; McMaster University, Canada

## Abstract

The insulin-like growth factor signalling pathway is an important regulator of skeletal muscle growth. We examined the mRNA expression of components of the insulin-like growth factor (IGF) signalling pathway as well as Fibroblast Growth Factor 2 (FGF2) during maturation of myotubes in primary cell cultures isolated from fast myotomal muscle of Atlantic salmon (*Salmo salar*). The transcriptional regulation of IGFs and IGFBP expression by amino acids and insulin-like growth factors was also investigated. Proliferation of cells was 15% d^−1^ at days 2 and 3 of the culture, increasing to 66% d^−1^ at day 6. Three clusters of elevated gene expression were observed during the maturation of the culture associated with mono-nucleic cells (*IGFBP5.1* and *5.2*, *IGFBP-6*, *IGFBP-rP1*, *IGFBP-2.2* and *IGF-II*), the initial proliferation phase (*IGF-I*, *IGFBP-4*, *FGF2* and *IGF-IRb*) and terminal differentiation and myotube production (*IGF2R*, *IGF-IRa*). In cells starved of amino acids and serum for 72 h, *IGF-I* mRNA decreased 10-fold which was reversed by amino acid replacement. Addition of IGF-I and amino acids to starved cells resulted in an 18-fold increase in *IGF-I* mRNA indicating synergistic effects and the activation of additional pathway(s) leading to IGF-I production via a positive feedback mechanism. *IGF-II*, *IGFBP-5.1* and *IGFBP-5.2* expression was unchanged in starved cells, but increased with amino acid replacement. Synergistic increases in expression of *IGFBP5.2* and *IGFBP-4*, but not *IGFBP5.1* were observed with addition of IGF-I, IGF-II or insulin and amino acids to the medium. IGF-I and IGF-II directly stimulated *IGFBP-6* expression, but not when amino acids were present. These findings indicate that amino acids alone are sufficient to stimulate myogenesis in myoblasts and that IGF-I production is controlled by both endocrine and paracrine pathways. A model depicting the transcriptional regulation of the IGF pathway in Atlantic salmon muscle following feeding is proposed.

## Introduction

Insulin-like growth factors (IGFs) and associated signaling pathways play a central role in regulating skeletal muscle growth in mammals [1], [2]. The expansion of muscle fibres in length and diameter is associated with net protein synthesis and nuclear accretion from a population of proliferating myogenic precursor cells [3], [4]. IGF-I and IGF-II promote growth by stimulating proliferation [5] and differentiation [6]–[8]. The IGF system includes the hormones IGF-I, IGF-II, their corresponding receptors and the IGF binding proteins (IGFBPs). Circulating IGFs are present in serum and are primarily secreted from the liver under the control of the pituitary/growth hormone axis [2], however both hormones are also synthesised locally in numerous tissues including skeletal muscle [9].

Stimulation of the cellular responses to IGF-I are mediated through the IGF-I receptor via the PI3K/AKT/mTOR pathway [8], with the availability of IGFs regulated by the insulin-like-growth factor binding proteins (IGFBPs). The IGFBPs are a family of secreted proteins with high affinity for IGFs with 6 distinct genes characterised in several mammalian species [10]–[12]. IGFBPs are expressed in different tissues throughout development and may protect IGFs from proteolytic degradation. As the IGFBPs have a higher affinity for the IGF ligand than its corresponding receptor, they regulate the availability of IGF-I to bind with its receptor [13]. The presence of certain domains in IGFBPs can facilitate binding to proteins such as cell surface proteins and proteins present in the extracellular matrix which results in reduced affinity of IGFBPs for IGF-I [14], [15]. Proteolytic degradation of IGFBPs by specific proteases also results in release of IGF-I [14], [16] thereby targeting IGF-I to particular tissues, and to particular cell types within those tissues. Different IGFBPs are known to stimulate and inhibit IGF stimulated growth processes in different tissues and developmental stages. For example, IGFBP-5 regulates the differentiation of C2C12 myoblasts through binding to IGF-II [17], whereas IGFBP-2 inhibits IGF-I stimulated cell proliferation and DNA synthesis, and is considered a negative regulator of growth [18]. IGF stimulation of processes such as differentiation can also be affected by the presence or absence of other growth factors. For example, basic fibroblast growth factor (bFGF) affects IGF signalling by inhibiting differentiation [19]–[21] and promoting cell proliferation [22]. In addition to stimulation by IGFs, the PI3K/AKT/mTOR pathway has recently been shown to be nutritionally regulated, in that mTORC1 phosphorylation is dependant on amino acid availability [23]. The amino acid leucine is unique in its ability to stimulate phosphorylation of mTORC1 [24] but is dependent on the preloading of cells with glutamine to facilitate leucine entry [25]. Several amino acid signalling pathways have now been identified including vps34 [26], recombination activating proteins (Rags) [27], [28] and the ste-20 related mitogen-activated protein kinase kinase kinase kinase 3 (MAP4K3) [29].

Teleosts are ectotherms resulting in major differences in metabolic regulation compared to mammals. For example, after correcting for body size and depending on the temperature the specific dynamic action can be 2–10 days, compared to a few hours in mammals [30]. In addition many fish species undergo long periods of fasting in the winter or during spawning migrations during which time skeletal muscle wasting occurs as part of a natural seasonal cycle [31]–[33]. It is therefore highly likely that the regulation of muscle mass shows some differences compared to mammals although the physiological mechanisms have yet to be investigated in detail.

The genomes of teleost fish contain multiple copies of many genes compared to mammals. This is because subsequent to divergence from a common ancestor to the tetrapods, teleosts underwent whole genome duplication (WGD), 320–350 million years ago, and rediploidisation with approximately 15% of the duplicated genes retained in extant species [34], [35]. Following the WGD in basal teleosts, an additional duplication event occurred 10–25 million years ago in the salmonid lineage [36] resulting in the presence of up to four salmonid paralogues for each mammalian gene. Of the duplicated genes, 50% may have subsequently been lost from the salmonid genome [37], with retained paralogues able to undergo sub-functionalisation leading to altered expression patterns [38]. The retention or loss of gene paralogues following WGD is lineage specific [38]. For example, there is one IGFBP-6 paralogue in Atlantic salmon and two in the zebrafish genome, whereas both species have retained two IGFBP-2 paralogues [39]–[41].

We have previously characterised the IGF signalling pathway in Atlantic salmon (*Salmo salar* L.) identifying paralogues of IGFBP-2, IGFBP-5, and IGF-I receptor [40]. In response to feeding, we observed biphasic increased expression of IGF-I, early increased expression of one IGFBP-5 paralogue and constitutive increased expression of IGFBP-4. The aim of the present study was to use a primary cell culture derived from fast skeletal muscle of Atlantic salmon to investigate the transcriptional regulation of the IGF components during myogenesis and obtain an insight into the roles of gene paralogues. Manipulative experiments, involving amino acid and serum withdrawal followed by addition of amino acids, recombinant IGF-I, IGF-II and insulin in various combinations, were used to investigate the nutritional and hormonal regulation of the IGF-system.

## Methods

### Ethics Statement

All experiments were approved by the Animal Welfare Committee of the University of St Andrews and fish were humanely killed following Schedule 1 of the Animals (Scientific Procedures) Act 1986 (Home Office Code of Practice. HMSO: London January 1997).

### Isolation of myogenic satellite cells

Fish used for cell culture were maintained in 1 m tanks at 10.8 C, and fed a commercially available fish diet (Ewos Innovation). Myosatellite cells were isolated from juvenile Atlantic salmon (*Salmo salar* L) 30±6 g (mean ±s.d., N = 10 per preparation) using a method similar to that previously described [42]. Fast myotomal muscle was dissected under sterile conditions and placed in extraction media consisting of Dulbecco's modified eagle's media (DMEM) 9 mM NaHCO_3_, 20 mM HEPES (pH 7.4) with 15% (v/v) horse serum and 1 x antibiotics (100 units/ml penicillin G, 100 µg/ml streptomycin sulfate, 0.25 µg/ml amphotericin B) (Sigma, Gillingham, Dorset, UK) at a ratio of 1 gram of muscle per 5 ml extraction media. The tissue was then minced with a sterile scalpel before centrifugation at 300 g for 5 min, and two washes with DMEM without horse serum. The muscle pieces were digested with collagenase (0.2% m/v in DMEM, Type 1a, Sigma, Gillingham, Dorset, UK) for 70 min at room temperature in the dark, before centrifugation at 300 g for 5 minutes. The resulting pellet was washed twice with DMEM before being passed through a pipette repeatedly to separate cells.

Samples were further digested with trypsin (0.1 m/v % in DMEM) for 20 minutes at room temperature. The resulting cell suspension was centrifuged (300 g, 1 min). The supernatant was poured into 20 x vol of extraction media containing serum to inhibit trypsin activity. The pellet was further digested by a second treatment with trypsin for 20 min at room temperature, before centrifugation at 300 g 1 min. The supernatant was poured into 20 x volume of extraction media. The extraction media containing the cell suspension was centrifuged at 300 g for 20 min. Cell pellets were re-suspended in 30 ml of basal medium before mechanical trituration through 10 ml and 5 ml pipettes until cells were separated. The cell suspension was then passed through 100 µm and 40 µm nylon cell strainers (BD Biosciences San Jose, CA, USA) and centrifuged 20 min 300 g. The cells were resuspended in basal media, cell number determined using a hymaecytometer, and then diluted to give approximately 1.5×10^6^ cells/ml.

### Cell culture

All cell culture methods were performed using Aseptic technique in a Microflow 2 Advanced biosafety cabinet (Bioquell Ltd, Andover, UK). 6 well cell culture plates (Greiner Bio-One Ltd, Stroudwater, UK) were treated with a 100 ug/ml poly-lysine solution (Sigma, Gillingham, Dorset, UK) at 4 µg/cm^2^ for 5 minutes at room temperature, then aspirated before 2 washes with sterile water and allowed to air dry. 1 ml of laminin (Sigma, Gillingham, Dorset, UK) in DMEM at 20 µg/ml was applied to each well and incubated at 18°C overnight prior to plated cells. Cell culture was performed using complete medium (DMEM, 9 mM NaHCO3, 20 mM HEPES (pH 7.4), supplemented with 10% foetal calf serum (Sigma) and 1 x antibiotics (Sigma, Gillingham, Dorset, UK) which was changed daily.

### Cell starvation and treatments

Cells were grown until day 9, and then washed once with amino acid deprived (starve) media (Earle's balanced salt solution, supplemented with 9 mM NaHCO3, 20 mM HEPES (pH 7.4), 2 g/l glucose, supplemented with 1 x vitamins, 1 x antibiotics), and then grown for 72 h in starve media. Control cells were grown in complete media. RNA was extracted from the cells 0, 6, 12, 24, 36, 48 and 72 h following treatment.

Cells starved for 72 h were then grown for 24 h in either starve media (control), starve media supplemented with IGF-I (recombinant salmon protein, GroPep, Adelaide, Australia) (100 ng/ml) or IGF-II (recombinant salmon protein, GroPep, Adelaide, Australia) (100 ng/ml), amino acid media (DMEM, 9 mM NaHCO3, 20 mM HEPES (pH 7.4), 1 x antibiotics), amino acid media with IGF-I (100 ng/ml), IGF-II (100 ng/ml) or IGF-I + IGF-II (100 ng/ml each), and amino acid media with 1 uM insulin (Sigma, Gillingham, Dorset, UK). RNA was extracted from cells 0, 3, 6, 12 and 24 h following treatment.

### Immunofluorescence of culture cells

Cells were grown on glass coverslips treated with poly-L-lysine and laminin as described above. Samples were washed 2 x in PBS, fixed in 4% (m/v) paraformaldehyde for 20 min at room temperature, washed 2×5 min in PBS, permeabilised with 0.2% triton X-100 PBS for 5 minutes, washed 2 x in PBS and then blocked in 5% NGS, 1.5% BSA, 0.1% triton X-100 PBS for 1 h at room temperature. All antibody steps were performed in PBST (1% BSA, 0.1% triton X-100 in PBS). Desmin antibody (Sigma, Gillingham, Dorset, UK) was diluted 1∶20 and anti-BrdU Alexa Flour 546 antibody diluted 1∶20 (Invitrogen, Carlsbad, CA, USA) in PBST and incubated overnight at 4°C, washed 3 x in PBS. For visualisation of desmin, a 1∶400 dilution of anti-rabbit Alexa Fluor 405 antibody (Invitrogen, Carlsbad, CA, USA) in PBST was incubated for 1 h at room temperature, and washed 3 x in PBS. Cells were then counterstained for nuclei with Sytox green (Invitrogen, Carlsbad, CA, USA) as per manufacturer's recommendations. Cells were imaged using a Leica TCS SP2 confocal microscope.

### Cell proliferation analysis

Cell proliferation rates were calculated by culturing cells in the presence of a 1∶100 dilution of BrdU (Invitrogen, Carlsbad, CA, USA) in complete media (Invitrogen, Carlsbad, CA, USA) for 8 h on sequential days. BrdU positive cells and sytox stained nuclei were visualised as described above, counted manually and the ratio of BrdU/sytox stained cells used to calculate the number of cells proliferating each day during an 8 h window. Cells were counted from 3 fields of view (0.56 mm^2^, 20× magnification) from 3 separate cultures.

### Quantitative real time PCR experiments

The following procedures were performed in order to comply with the Minimum Information for Publication of Quantitative Real-Time PCR experiments MIQE guidelines [43].

### RNA extraction and cDNA synthesis

RNA was immediately extracted from duplicate wells of 3 separate cell cultures. RNA extraction and genomic DNA removal was performed using a RNeasy plus kit (Qiagen Inc., Chatsworth, CA, USA) as per manufacturer's recommendations. RNA was concentrated by ethanol precipitation and quantified using a NanoDrop 1000 spectrophotometer (Thermo Fisher Scientific, Waltham, MA, USA). Only RNA with an A260/280 ratio between 1.8 and 2.1 and an A260/230 above 2.0 was used for cDNA synthesis. For samples where enough RNA was obtained (excludes day 2), the integrity of the RNA was confirmed by gel electrophoresis. Residual genomic DNA was removed using the genomic DNA wipeout buffer included in the Quantitect reverse transcription kit (Qiagen Inc., Chatsworth, CA, USA). 800 ng of RNA was reverse transcribed into cDNA for 30 min at 42°C using a Quantitect reverse transcription kit (Qiagen Inc., Chatsworth, CA, USA) as per manufacturer's recommendations. To check for contaminating genomic DNA, a control reaction was performed which lacked the reverse transcriptase enzyme (–RT).

### Quantitative PCR

qPCR was performed using a Stratagene MX3005P QPCR system (Stratagene, La Jolla, CA, USA) with Brilliant II SYBR (Stratagene, La Jolla, CA, USA). Each qPCR reaction mixture contained 7.5 µl 2 x Brilliant II SYBR green master mix (Surestart Taq DNA polymerase, 2.5 mM MgCl_2_, 6 µl cDNA), cDNA (80-fold dilution, 20-fold for IGF-I), 500 nM each primer and RNase free water to a final volume of 15 µl. Amplification was performed in duplicate in 96 well plates (Stratagene, La Jolla, CA, USA) with the following thermal cycling conditions: initial activation 95°C for 10 minutes, followed by 40 cycles of 15 s at 95°C, 30 s at 60°C, and 30 s at 72°C. Control reactions included a no template control (NTC) and -RT. Dissociation analysis of the PCR products was performed by running a gradient from 60 to 95°C to confirm the presence of a single PCR product. Products were also sequenced to confirm identity. A dilution series made from known concentrations of plasmid containing the PCR inserts was used to calculate absolute copy numbers for each of the genes examined.

Standards for calculating absolute copy number for each gene were prepared by cloning the PCR product from each primer pair into a T/A pCR4-TOPO vector (Invitrogen, Carlsbad, CA, USA) and transformation of chemically competent TOP10 *Escherichia coli* cells (Invitrogen,. Carlsbad, CA, USA). Individual colonies were grown and plasmids purified using a Fastprep plasmid purification kit (Eppendorf, Hamburg, Germany). The concentration of each plasmid was calculated based on absorbance at 260 nm, and a dilution series produced for calculation of copy number via qPCR. For relative quantification of IGF receptor paralogues, the PCR efficiencies were calculated from a dilution series (1/20, 1/40, 1/80, 1/160, 1/320, 1/640) of pooled cDNA samples.

### Primer design

Many of the primers used for qPCR have previously been described [40] and are listed in table 1. Primers were designed using NetPrimer (Premier BioSoft) to have Tm of 60°C, and where possible, to cross an exon-exon junction to avoid amplification of genomic DNA. To determine exon-intron junction sites, genomic sequences for orthologous genes from *Danio rerio*, *Gasterosteus aculeatus*, *Oryzias latipes*, *Takifugu rubripes* and *Tetraodon nigroviridis* were retrieved from Ensembl (http://ensembl.org/), and compared to the *Salmo salar* cDNA sequences using the Spidey software tool (http://www.ncbi.nlm.nih.gov/spidey/). Primers were used at a final concentration of 500 nM.

**Table 1:** qPCR primer sequences, PCR efficiencies, correlation coefficients of standard curves, amplicon size and melting temperature.

Gene	Forward and Reverse primer (5′-3′)	E (%)	r^2^	size (bp)	Tm ° C	Accession number
*EF1α*	F: GAATCGGCTATGCCTGGTGAC R: GGATGATGACCTGAGCGGTG	96.0	0.998	141	86.0	BG933853
*Ppia*	F: CATCCCAGGTTTCATGTGC R: CCGTTCAGCCAGTCAGTGTT	96.4	0.998	203	85.9	DY727143
*Hprt1*	F: CCGCCTCAAGAGCTACTGTAAT R: GTCTGGAACCTCAAACCCTATG	92.1	0.996	255	81.8	EG866745
*FGF2*	F: ATAAGCTTCAACTCCAGGCGACC R: AGCATTCATCTGTTGTCCGTCTC	102.1	0.990	230	81.7	GE794494
*IGF-I*	F: CCTGTTCGCTAAATCTCACTTC R: TACAGCACATCGCACTCTTGA	102	0.995	226	81.7	EF432852
*IGF-II*	F: GAAAACACAAGAATGAAGGTCAA R: CCACCAGCTCTCCTCCACATA	94.0	0.996	127	82.7	EF432854
*IGF-IRa*	F: GGGGCTCTCCTTCTGTCCTA R: AGAGATAGACGACGCCTCCTA	97.2	0.995	175	85.9	EU861008
*IGF-IRb*	F: CTAAATCTGTAGGAGACCTGGAG R: GGTTAGCCACGCCAAATAGATCC	100.0	0.996	138	86.3	EU861006
*IGF2R*	F: CTTCATCCACGCTCAGCAG R: ACCCTGGGCCGTGTCTAC	96.0	0.998	168	84.3	CX325971
*IGFBP-1*	F: AGGACCAGGGACAAGAGGAAG R: CTGTTCCACCAGTTTCTTGC	98.2	0.995	154	82.3	EF432856
*IGFBP-2.1*	F: CGGTGAGGAAGGCCACTAAGG R: ATATCACAGTTGGGGATGT	95.0	0.998	249	85.3	EF432858
*IGFBP-2.2*	F: TTCCATGATAACAGGGGACCAG R: GACCGTGGGTGGACATGTGG	94.7	0.994	108	79.6	EF432860
*IGFBP-4*	F: ACTTCCATGCCAAGCAGTGC R: GGTCCCATCCTCACTCTCTC	95.6	0.993	164	87.5	EU861007
*IGFBP-5.1*	F: ATCACGGAGGACCAACTGC R: TGCTTGTCAATGGGTAGTGG	101.0	0.997	169	86.4	EF432862
*IGFBP-5.2*	F: TTCTCCAGAGGAAGCTATGTTAG R: TCAAGGCTGCTGACAGAGTG	96.7	0.996	170	86.3	EU861009
*IGFBP-6*	F: GCTGCGTGCCTCTTCCTCA R: TTACGGCAGGGTGCCTTTTC	101.0	0.998	159	86.1	EF432864
*IGFBP-rP1*	F: GAAGTGTGTGGCTCCGATG R: GTTTTCCGCTGGTGACCTTCT	103.2	0.997	249	84.8	EF432866
*Rps29*	F: GGGTCATCAGCAGCTCTATTGG R: AGTCCAGCTTAACAAAGCCGATG	96.1	0.998	167	84.7	NM_001139600
*Rpl13*	F: CGCTCCAAGCTCATCCTCTTCCC R: CCATCTTGAGTTCCTCCTCAGTGC	98.9	0.998	79	81.8	BT043698

### Data analysis

The stability of reference genes for Atlantic salmon myogenic cell culture has been previously described [44]. Data were analysed using geNorm [45] using the geometric average of Elongation factor 1 alpha (*EF1-α*), hypoxanthine phosphoribosyl transferase 1 (*Hprt1*) and peptidyl prolyl isomerase A (*Ppia*) with the normalised gene expression values displayed as arbitrary units (A.U.). Input data for geNorm was absolute values derived from a plasmid standard curve. Melting temperatures, efficiency values and r^2^ values for standard curves are given in table 1. Relative expression levels of *IGF-IR* paralogues were calculated with *Ppia* as reference gene using the Q-gene software [46]. Ribosomal protein S29 (*Rps29*) and ribosomal protein L13 (*Rpl13*) were the most stable genes in starved and treated cells and so the geometric average of these two genes were used for normalisation of starved and treated cells gene expression data. Statistical analysis was performed with Minitab (Minitab Inc). When data conformed to parametric assumptions, ANOVA using Fisher's individual error post hoc test was used to identify significant differences. When parametric assumptions were not met, Kruscal Wallis test was used. Correlations in gene expression were calculated using Pearson's correlation, and when data where not normally distributed, by Spearman rank correlation. Differences in gene expression are indicated by different letters on the graphs. Hierarchical clustering analysis of gene expression data was performed using Permutmatrix [47].

## Results

### Myogenic cell culture

**Figure 1:**
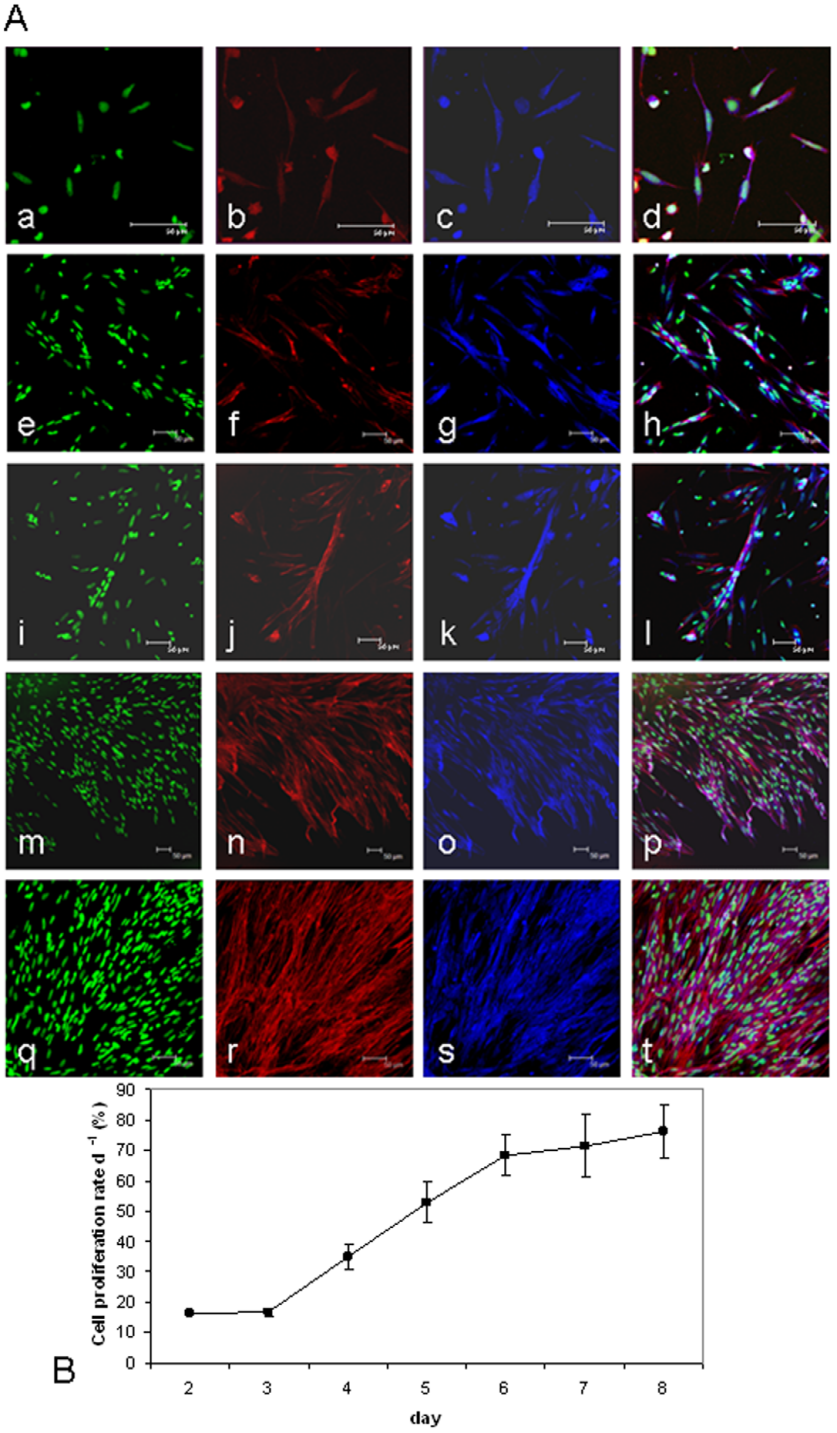
Atlantic salmon myogenic cell culture phenotype and cell proliferation rates. (A) Growth and differentiation of myogenic cells extracted from *S salar* fast myotomal muscle at 2 d (a–d), 5 d (e–h), 8 d (i–l), 11 d (m–p) and 14 d (q–t). Nuclei stained with sytox green (a,e,i,m,q) and actin counterstained with phalloidin (b,f,j,n,r) confirmed the presence of multinucleated myotubes. Myogenic cells were also identified by positive desmin staining (c,g,k,o,s). The combined images are shown in the overlay (d,h,l,p,t). Scale bars represent 50 µm. (B) Proliferation rate of cultured myogenic cells calculated as the proportion of BrdU positive nuclei to sytox green stained nuclei following an 8 h exposure to BrdU (mean ± S.E. n = 9).

Cell cultures were visualised using confocal microscopy and the phenotype of cells determined at 2 d, 5 d, 8 d, 11 d and 14 d (Fig. 1). The myogenic nature of the cell culture was confirmed by the presence of multi-nucleated myotubes visualised by nuclei stained with sytox green (Fig. 1A a,e,i,m,q), Alexa Fluor 568-phalloidin stained actin filaments (Fig. 1A b, f, j, n, r) and the presence of the myogenic marker desmin (Fig. 1A c,g,k,o,s). At 2 d, all cells were mononucleic (Fig. 1a–d), which then fused to form small myotubes at 5 d (Fig. 1A e–h) and 8 d (Fig. 1A i–l) and then as the culture progressed, longer myotubes (Fig. 1m–p) and sheets of longer multi-nucleated myotubes at 14 d (Fig. 1A q–t).

### Cell proliferation

Initially, cells proliferated at a rate of 15%±0.32% d^−1^ (n = 9 Mean ± S.E.), which then increased linearly from days 3 to 6 (r^2^ = 0.99), reaching 66%±6.86% d^−1^ (n = 9 Mean ± S.E.) (Fig. 1B). Proliferation rates remained at a constant level until 8 d, after which cells continued to proliferate, but accurate measurement became impractical.

### Expression of IGF-I, IGF-II and FGF2 and IGF receptors

**Figure 2:**
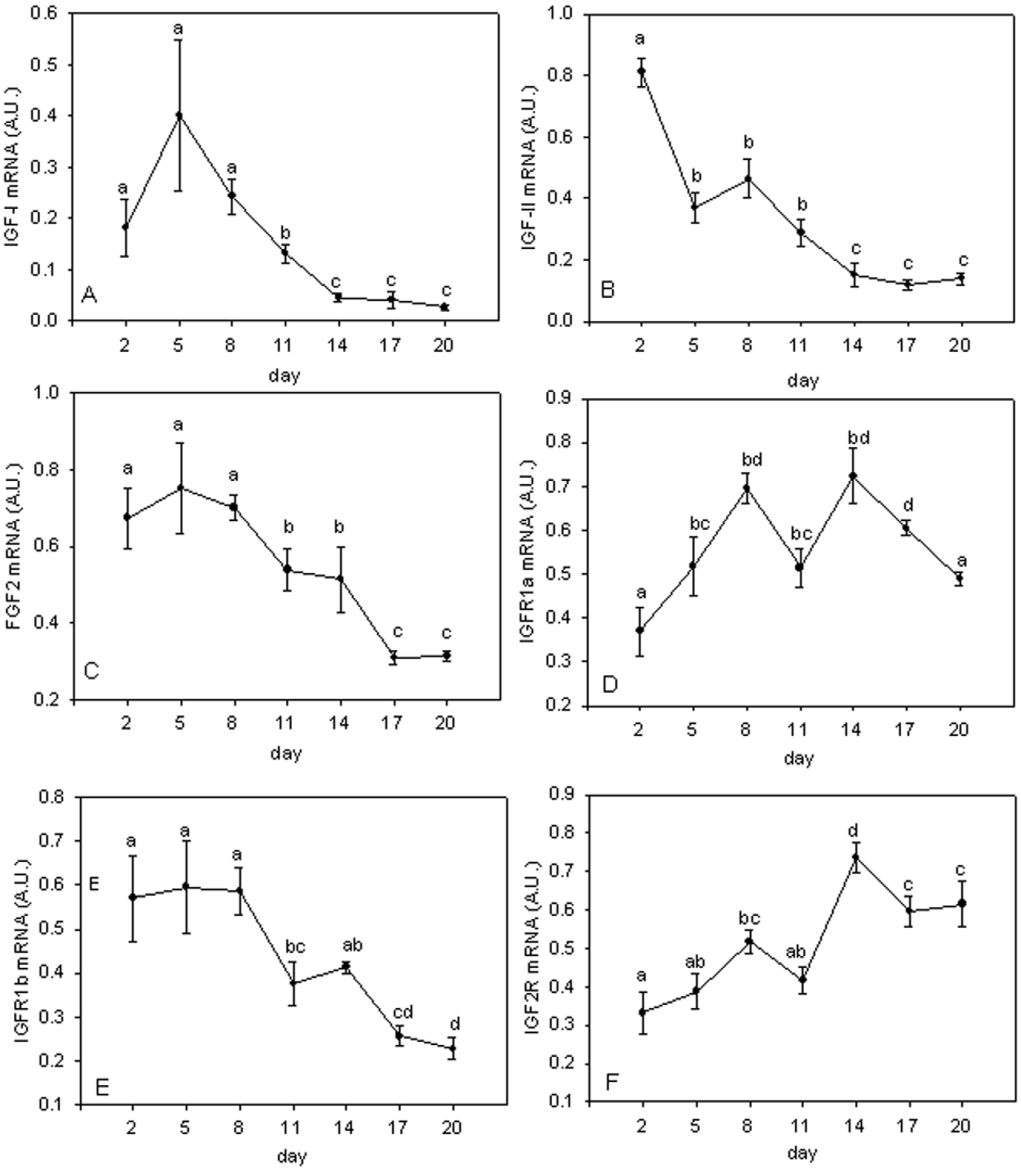
Gene expression profiles for *IGF-I, IGF-II, FGF2, PCNA, IGFR1a, IGFR1b* and *IGF2R* during myotube maturation. Expression profiles for *IGF-I* (A), *IGF-II* (B), *FGF2* (C), *PCNA* (D), *IGF-IRa* (E), *IGF-IRb* (F) and *IGF2R* (G) in Atlantic salmon myogenic cell culture at 2 d, 5 d, 8 d, 11 d, 14 d, 17 d and 20 d. Values represent mean ± S.E., 6 samples per point from three cultures. Significant differences between means are indicated by different letters.

*IGF-I* expression increased to a peak at 5 d of culture and then decreased by 14 d to a level 15-fold less than at 5 d (Fig. 2A). *IGF-II* expression was highest at 2 d in mononuclear cells and was then downregulated before reaching a steady state expression at 14 d, 5-fold lower than peak levels (Fig. 2B). Fibroblast growth factor 2 (*FGF2*) expression showed relatively high expression until 8 d of culture, but then, like *IGF-I* and *II*, declined as the culture matured (P<0.05) (Fig. 2C).

The two paralogues for *IGF-IRA* had distinct expression patterns. *IGF-IRa* expression increased during maturation of the culture showing peak values at 8 d and 14 d (P<0.05, Fig. 2D). In contrast, *IGF-IRb* expression was high until 8 d and then decreased 2.3-fold at 17 d (P<0.05, Fig 2E). *IGF-IRa* mRNA levels were on average 100 times more abundant than that of IGF-IRb (P<0.001, data not shown). *IGF2R* mRNA increased during maturation of the culture with peak levels at 14 d (P<0.01, Fig. 2F).

### Expression of IGFBPs

**Figure 3:**
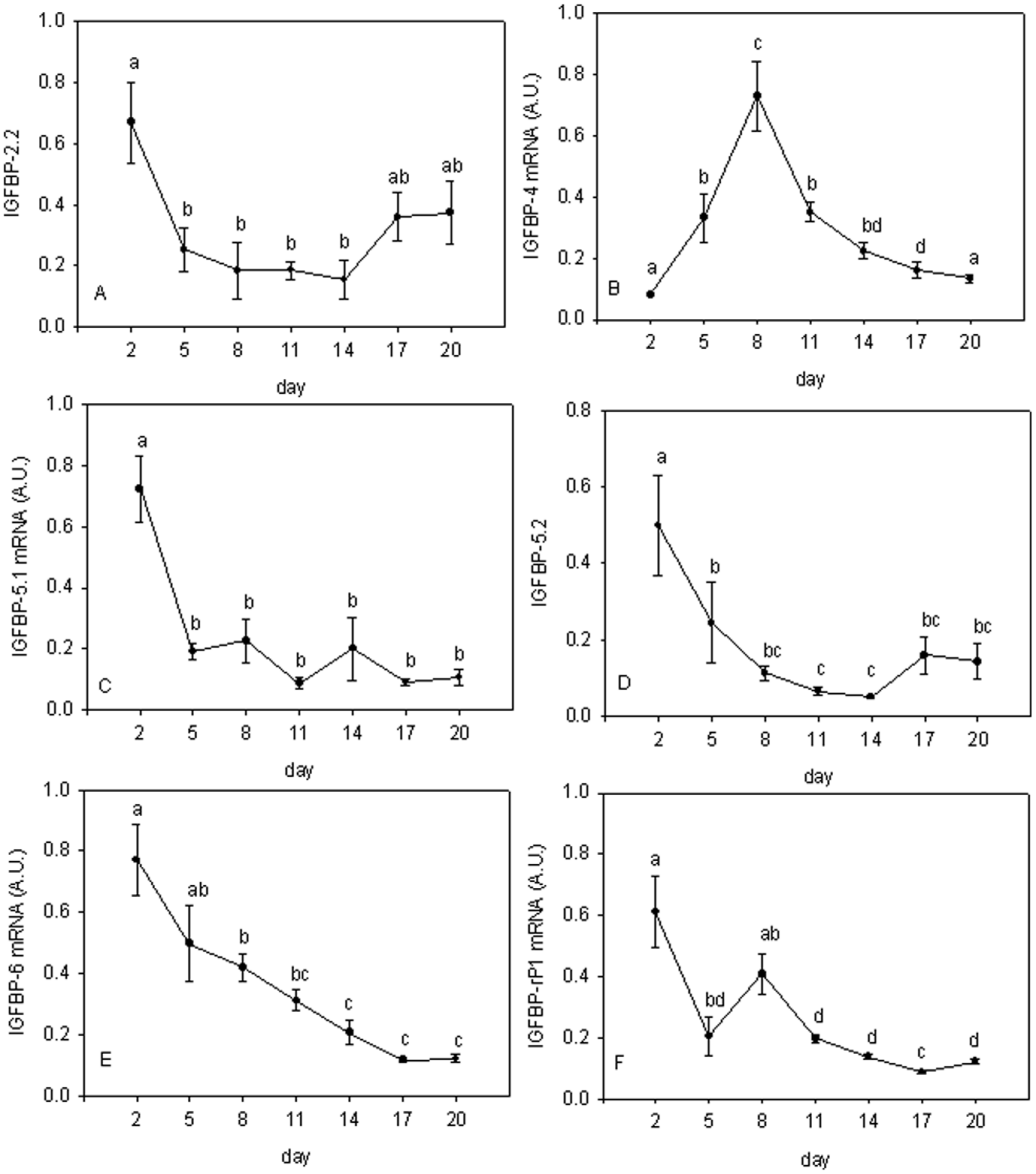
Gene expression profiles for *IGFBPs* during myotube maturation. Expression profiles for *IGFBP-2.1* (A), *IGFBP-4* (B), *IGFBP-5.1* (C), *IGFBP-5.2* (D), *IGFBP-6* (E) and *IGFBP-rP1* (F) in Atlantic salmon myogenic cell culture at 2 d, 5 d, 8 d, 11 d, 14 d, 17 d and 20 d. Values represent mean ± S.E., 6 samples per point from three cultures. Significant differences between means are indicated by different letters.

The mRNA levels for *IGFBPs 1* and *2.1* were below the limits of detection using qPCR. *IGFBP-2.2* expression was highest at 2 d corresponding to mononuclear cells, decreased 4-fold at 14d before increasing again in the later stages of the culture when syncytial sheets of myotubes had formed (P<0.05, Fig. 3A). *IGFBP-4* was present at relatively low levels at the early (2d) and late (17, 20 d) stages with a distinct 8-fold increase in expression at 8d (P<0.001, Fig. 3B). Expression of *IGFBP-5.1* (Fig. 3C), *IGFBP-5.2* (Fig. 3D) and *IGFBP-6* (Fig. 3E) were high in mononuclear cells (2d) then declined as the culture matured and the proportion of differentiated myotubes increased (P<0.01). *Insulin like growth factor binding protein-related protein-1* (*IGFBP-rP1*) showed peaks of expression at days 2 and 8 of the culture (Fig. 3F).

### Gene expression in starved cells

**Figure 4:**
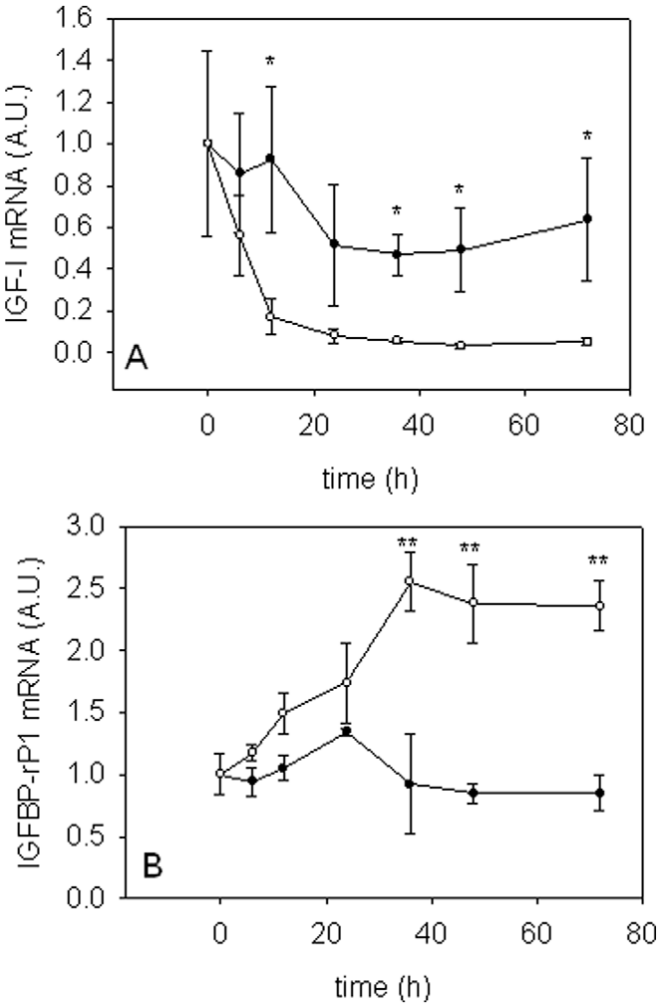
Gene expression profiles during serum deprivation. Expression profiles of *IGF-I* (A) and *IGFBP-rP1* (B) in 9 d myogenic cells in complete media (closed circle) or amino acid and serum deprived media (open circle). For ease of interpretation, values at time 0 h were transformed so that the mean value is equal to 1. Values represent mean ± S.E., 3 independent cell cultures. Significant differences in expression are indicated by an asterisk (* P<0.05, ** P<0.01).

Only two of the genes studied showed altered expression in cells deprived of amino acids and serum. *IGF-I* mRNA decreased significantly in starved cells within 12 h (P<0.05, 5-fold downregulation) and to levels more than 10-fold lower than in control cells by 72 h (P<0.05, Fig 4A). Steadily increasing expression of *IGFBP-rP1* was observed in starved cells, reaching levels 2.5-fold higher than in control cells (P<0.05, Fig. 4B).

### Gene expression in starved cells treated with amino acids and IGF-I and II

**Figure 5:**
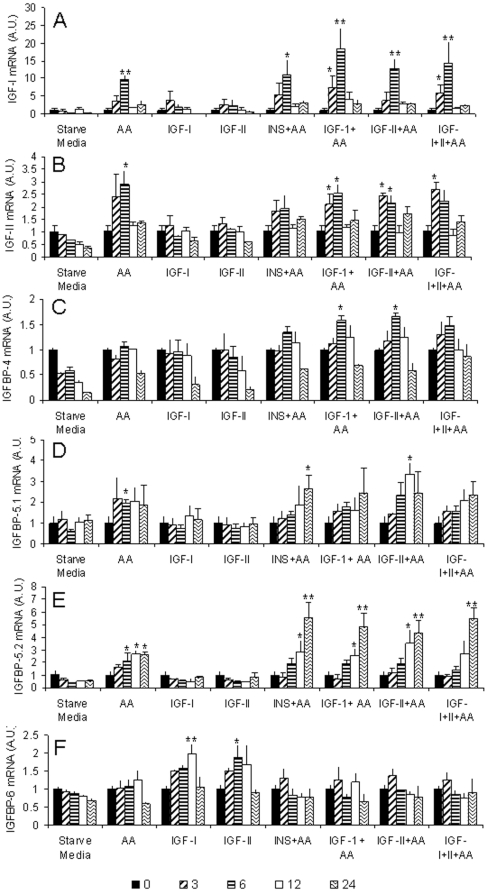
Analysis of stimulation of the IGF-signaling pathway by amino acids and IGFs. Expression profiles for *IGF-I* (A), *IGF-II* (B), *IGFBP-4* (C), *IGFBP-5.1* (D), *IGFBP-5.2* (E) and *IGFBP-6* (F) in day 9 myogenic cells starved for 72 h, then treated with various combinations of amino acids, IGFs and insulin or starve media (starve media  =  no amino acid or serum). cDNA was synthesised from RNA extracted 0, (black bar) 3 (diagonal stripe), 6 (horizontal stripe), 12 (white bar) and 24 h (sigmoidal stripe) following treatment. For ease of interpretation of the cell treatments, the 72 h starved cell values were transformed so that the mean value is equal to 1. Values represent mean ± S.E., 3 independent cell cultures. Changes in expression were considered significant if they were different to both the 72 h starved value and the control treatment. Significant differences in expression are indicated by an asterisk (* P<0.05, ** P<0.01).

Addition of amino acids to starved cells resulted in a 10-fold increase in *IGF-I* expression within 6 h (p<0.01), although this peak level of expression had fallen by 12 h (Fig. 5A). Treatment with IGF-I and IGF-II alone stimulated *IGF-I* mRNA expression at 3 and 6 h, however, the increase was only 2.5-3.5-fold and not statistically significant. IGF-I combined with amino acids leads to a synergistic response as an 18-fold increase was observed after 6 h when both stimuli were present (P<0.01, Fig. 5A). *IGF-II* mRNA increased 2 to 3-fold at 3-6 h in response to amino acid stimulus (P<0.05), but did not respond to IGFs (Fig. 5B).

Amino acid stimulation resulted in increased expression of *IGFBP-4* 6 h following treatment (P<0.05, Fig. 5C), but only when amino acids and IGFs or insulin were the combined stimulus. The two *IGFBP-5* paralogues increased in response to amino acid stimulation after 6 h (P<0.05), and were still elevated at 24 h (Figs. 5D and 5E). Expression of both paralogues did not change in the presence of IGF-I or IGF-II alone, however both paralogues increased expression with amino acid addition. Interestingly, for *IGFBP-5.2*, a synergistic effect was observed after 24 h when IGF-I, IGF-II or insulin were added in the presence of amino acids (P<0.01, Fig. 5E). Expression was increased from 2-fold for amino acid stimulation alone, up to 5-fold when amino acids were combined with either hormone. *IGFBP-6* was unique in being the only IGFBP to respond to IGF-I or IGF-II alone, with increased expression observed 12 h following stimulation (p<0.05, Fig. 5F). IGFBP-6 mRNA levels remained unchanged in the presence of amino acids.

Expression of *IGFBP-2.1* and *2.2* was below the limits of detection. For the remaining genes examined (*FGF2*, *IGF-IRa*, *IGF-IRb*, *IGF2R*, *IGFBP-rP1*) there were no significant changes in gene expression with the treatments.

### Expression relative to myogenic regulatory factors

**Figure 6:**
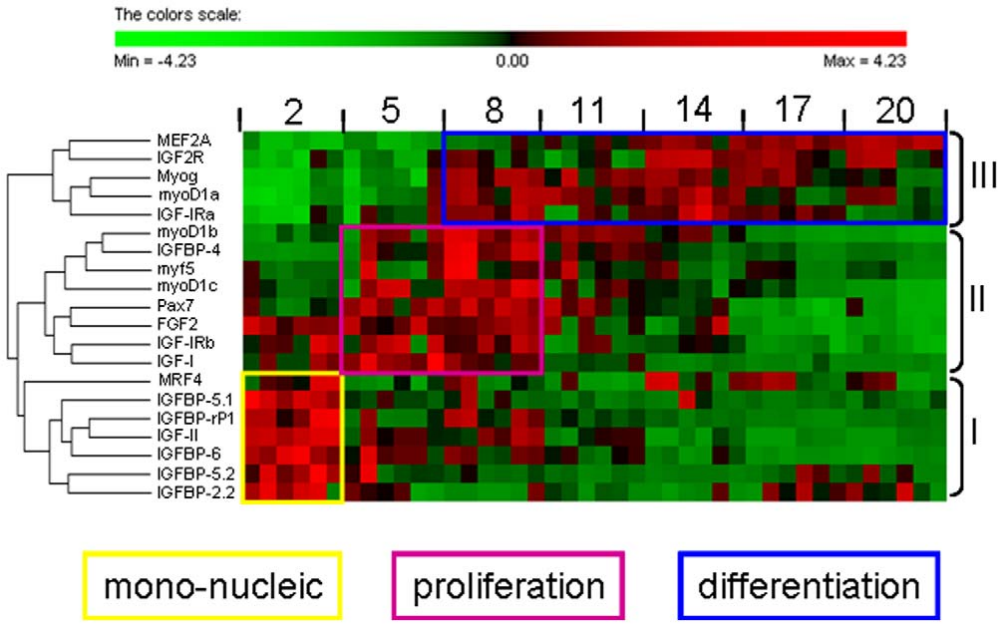
Heat map summary and hierarchical clustering of gene expression. Heat map summary and hierarchical clustering for MRFs, *MEF2A*, *pax7*, *FGF2* and components of the IGF signaling pathway during Atlantic salmon myogenic cell culture at 2 d, 5 d, 8 d, 11 d, 14 d, 17 d and 20 d. Rows are standardized to have mean 0 and standard deviation 1 so that red indicates high and green indicates low values.

We have previously examined the expression of the myogenic regulatory factors (MRFs) *myoD1a, 1b, 1c, myog, Myf5* and *MRF4, MEF2A* and a marker of myogenic progenitor cells (*Pax7*) using the same cultures [48]. To place the components of the IGF signalling pathway into a myogenic framework, we clustered their expression during myotube maturation with those of the *MRFs, MEF2A* and *Pax7*. A summary heat map and hierarchical clustering of gene expression patterns during the maturation of the culture is shown in Fig. 6.

A number of genes showed highly correlated expression patterns suggesting that they may be regulated by similar pathways. These included *myoD1b* and *IGFBP-4* (R = 0.88, P<0.0001, Fig. S1A), *IGFBP-4* and *myf5* (R = 0.84, P<0.0001 Fig. S1B) and *IGF-II* and *IGFBP-rP1* (R = 0.81, P<0.0001 Fig. S1C). Correlated patterns of gene expression patterns were observed in the individual cultures as well as in the overall analysis, demonstrating that the correlations are not an artefact of combining the data from three cultures.

## Discussion

Distinct phases of gene expression are apparent associated with mono-nucleic cells, the initial cell proliferation phase and terminal differentiation and myotube formation (Figs. 1A, 1B & 6). Cluster I comprised genes predominantly expressed in mono-nucleated cells and included *IGFBP-5.1* and *5.2*, *IGFBP-6*, *IGFBP-rP1*, *IGFBP-2.2*, *MRF4* and *IGF-II*. Cluster II comprised *myoD1b* and *1c*, *myf5*, *pax7*, *IGF-I*, *FGF2* and *IGF-IRb*, and had highest expression during the initial phase of cell proliferation. Cluster III contained genes that were predominantly expressed in differentiated cells and was comprised of *MEF2A*, *IGF2R*, *myog*, *myoD1a* and *IGF-IRa*.

Expression of IGF-I (Fig. 2A) and IGF-II (Fig. 2B) was higher at 2-8 d, than at 14-20 d when multinucleated myotubes predominated (Fig. 1A). This high expression at early time points may be necessary to start cell proliferation and for quiescent cells to enter the cell cycle. There was a transient increase in *IGF-II* expression at 8 d (Fig. 2B), which coincides with the maximal expression of *myog* (Fig. 6) and all three *myoD* paralogues (Fig. 6). In a NFB4 mutant muscle cell line, exogenous IGF-I and IGF-II induce expression of *myog*, and *myog* over-expression increases differentiation and *IGF-II* expression, showing IGF-II and myog can regulate each others transcription [49]. MyoD overexpression in CH3 10T1/2 cells induces IGF-II gene and protein expression as early events in differentiation, leading to activation of the IGF-I receptor and down stream signalling molecules such as AKT [50]. IGF-II is also known to be more potent in stimulating myogenesis than IGF-I, whereas the actions of IGF-I have been shown to delay differentiation [51].

During the early phase of cell culture, IGF-I signals through the ERK1/2 MAPK pathway, then through AKT at later time points [52]. The presence or absence of other growth factors such as basic fibroblast growth factor (bFGF), which can also signal via ERK 1/2 MAPK pathway [53], may attenuate the effects of IGFs [22] by inhibiting differentiation [19]–[21] and promoting myoblast cell proliferation [22]. High expression of *FGF2* (Fig. 2C) coincided with high expression of *IGF-I* (Fig. 2A) at early stages of the cell culture as cells began to proliferate (Fig. 1B), consistent with a role in cell proliferation. As cells underwent differentiation, indicated by the highest levels of *myog* expression at days 8–14 (Fig. 6), expression of *FGF2* decreased (Fig. 2C).

IGF signalling requires the IGF hormones to bind to the IGF-I receptors to initiate the signalling cascade that results in phosphorylation of AKT/mTOR. In Atlantic salmon, two paralogues of IGF-IR are found. IGF-IRa mRNA levels were on average 100-fold more abundant than IGF-IRb, which recapitulates their *in vivo* levels (Bower and Johnston, unpublished results). The two paralogues were also differentially expressed relative to each other (Fig. 2D and 2E), with IGFR1a mRNA levels increasing while IGFR1b decreased. The differences in expression suggest that the genes coding for the two receptors have acquired distinct cis regulatory elements since they were duplicated. IGF-I receptors in sea bream increase in number as myogenic cells differentiate [52], which may be important in the switch from MAPK to AKT signalling. Based on the expression we observed, IGF-IRa is most likely to play this role in the differentiation of Atlantic salmon myoblasts.

Expression of IGF2R increased throughout the culture, with highest levels observed in differentiated cells (Fig. 2F). The IGF2R has been demonstrated to target IGF-II for degradation via the lysosomal pathway [54]. However, recent findings suggest that the IGF2R may trigger a signalling cascade leading to cardiac muscle cell hypertrophy [55], the regulation of cell invasion and cell motility in human cancer cells [56], [57] and the binding of multiple ligands other than IGF-II [58], [59].

*IGFBP-2.2* expression was down regulated from 2 d onwards (Fig. 3A), consistent with IGFBP-2 being a negative regulator of growth [18]. IGFBP-4 has recently been shown to be a negative regulator of growth when overexpressed in zebrafish embryos [60]. We found *IGFBP-4* expression peaked at 8 d (Fig. 3B), coinciding with maximal myog expression (Fig. 6), with values 7-fold higher than in mono nuclear myoblasts, before declining at later stages of the culture (Fig. 3B). Interestingly, expression of *IGFBP-4* was highly correlated with *myoD1b*R = 0.88, p<0.0001, Fig. 6, Fig. S1A) and also *myf5* (R = 0.84, p<0.0001, Fig. 6, Fig. S1B) and thus may share common cis regulatory elements with these MRFs. We have previously reported that *IGFBP-4* was the only IGFBP constitutively upregulated in response to refeeding in Atlantic salmon *in vivo* [40], which combined with the expression pattern reported here, suggests that *IGFBP-4* is a promyogenic factor in Atlantic salmon.

Differentiation of C2C12 muscle cells is promoted by IGFBP-5 [61] and expression increases during differentiation of C2 myoblast cell line [62]. In contrast to these findings in mouse cell lines, we found expression of both *IGFBP-5* paralogues significantly decreased from 2 d, reaching minimal expression levels by 5 d for *IGFBP-5.1* (Fig. 3C) and 8 d for *IGFBP-5.2* (Fig. 3D). We have previously reported an increase in *IGFBP-5* expression as an early response to feeding, similar to findings in rainbow trout [63]. From these results, a plausible hypothesis would be that IGFBP-5 plays a role in stimulating MPCs to enter the cell cycle in Atlantic salmon.

Cell proliferation has been shown to be inhibited by IGFBP-6 in many mammalian cell types [64]. Furthermore, IGFBP-6 inhibits growth in zebrafish embryos [41]. Consistent with these findings, expression of *IGFBP-6* significantly decreased (Fig. 3E) as cell proliferation increased (Fig. 1B). Mammalian IGFBP-6 has a 10–100 fold higher affinity for IGF-II than IGF-I [65], so we suggest that downregulation of *IGFBP-6* may increase the availability of IGF-II to other binding proteins required for differentiation.

### Stimulation of gene expression by amino acids and IGFs

*IGF-I* mRNA expression is modulated by nutrition in a number of fish species [40], [63], [66]. Consistent with these findings, we observed decreased IGF-I expression within 12 h of amino acid and serum withdrawal, by as much as 10-fold after 72 h (Fig. 4A). This reduction in *IGF-I* mRNA levels was rapidly reversed by addition of amino acids, with expression increasing 3- and 9-fold after 3 and 6 h respectively (Fig. 5A). Amino acids and IGFs had a synergistic effect on *IGF-I* expression, suggesting that separate signalling pathways have been activated, or that different components of the same pathway are responsive to amino acids and IGFs. It is very interesting that we observed an increase in *IGF-I* mRNA expression by IGF-I stimulation, as IGF-I downregulates *IGF-I* mRNA in C2C12 myoblasts [67]. Paracrine/autocrine production of IGF-I appears to be more important than circulating levels in stimulating muscle growth [68]–[71]. It is therefore possible that IGF-I mRNA expression stimulated by IGF-I peptide in teleosts could be an important factor in the indeterminate growth of teleosts in comparison to mammals. The transcriptional activation of IGF-I by amino acids also suggests that local IGF-I production in fast muscle can occur independently of growth hormone, insulin and IGFs. Amino acid availability, and in particular, the essential amino acid leucine is required for activation of mTORC1 in mammals [23], [24]. Amino acid stimulation could occur through an amino acid sensing signalling pathway regulated by either vps34 (a class 3 PI3K) [26], recombination activating proteins (Rags) [27], [28] or the ste-20 related mitogen-activated protein kinase kinase kinase kinase 3 (MAP4K3) [29] leading to activation of mTORC1 [72].

Although IGF-II mRNA expression did not change by amino acid and serum withdrawal, mRNA levels increased specifically in response to amino acids, similar to mammals [73]. We have recently shown that amino acid and serum starvation of Atlantic salmon myogenic cells results in cells withdrawing from the cell cycle and entering a quiescent state [48] and that upon stimulation by amino acids they enter the cell cycle. The observed increase in IGF-II mRNA at 3 and 6 h suggests that increased expression may be necessary for cells to enter the cell cycle. This is consistent with the high expression observed at 2 to 8 d in the cell culture (Fig. 2B).

Amino acids alone were able to stimulate the expression of both IGFBP-5 paralogues (Fig. 5D and 5E) and a synergistic effect was observed for *IGFBP-5.2* expression when IGFs or insulin were also present. This suggests that two separate pathways need to be stimulated to achieve maximum transcriptional activity, similar to the synergism between amino acids and insulin in phosphorylation of S6K1 in trout [74]. Interestingly, the synergistic effect was observed for all three hormones, suggesting that signalling occurs through insulin receptor substrate-1 (IRS-1), which is a common substrate for both IGF-I and insulin receptors, leading to activation of AKT/mTOR pathway [75]. Signaling through IRS-1 and the PI3K/AKT/mTOR pathway regulates IGF-I induced IGFBP-5 expression in smooth muscle cells [76] and could converge with the amino acid signaling pathway on mTORC1, where recently, phosphorylation of S1261 has been demonstrated to occur through insulin signaling in an amino acid dependent manner [23]. The lack of a synergistic response for *IGFBP-5.1* suggests that the promoters of the two paralogues contain different cis regulatory elements responsible for their different expression responses. The synergistic effect of IGFs and amino acids on *IGFBP-5.2* expression could explain the differences in gene expression observed in Atlantic salmon fed to satiation after a period of reduced feed intake, where only *IGFBP-5.2* showed a positive transcriptional response [40]. The expression profile of both *IGFBP-5* paralogues was temporally distinct to the IGFs and other binding proteins, with upregulation by amino acids combined with IGFs only seen at 12 and 24 h post stimulation. This suggests that *IGFBP-5* lies further downstream in the signalling pathway and needs other cofactors to be activated other than those stimulated directly by amino acids and IGFs.

IGFBP-4 showed a more restricted expression pattern, and was only increased when amino acids were present with IGFs or insulin (Fig. 5C). The requirement of both stimuli to be present for increased expression, combined with the peak expression coinciding with maximal myog expression in developing myotubes (Figs. 1 and 3B) suggests an important role for IGFBP-4 in both nutritional and myogenic processes.

IGFBP-6 was unique amongst the binding proteins in being stimulated by IGFs alone, and the increased expression appears to be inhibited by the presence of amino acids (Fig. 5F). As IGFBP-6 has been considered a negative regulator of cell proliferation [64], a plausible hypothesis based on the observed expression pattern is that after ingested amino acids have been metabolised and become depleted, *IGFBP-6* expression is increased, due to the presence of locally produced IGF-I and II, and its translational product sequesters available IGFs, thereby inhibiting any positive feedback mechanisms. This could be a mechanism to tightly control the availability of IGF-II, as *IGF-II* expression was only increased by amino acids (Fig. 5B).

It is striking that we observe distinct temporal patterns of expression for each of the binding proteins within a 24 h period upon stimulation by the various treatments. This could have important implications for many studies examining the function of the IGFBPs. For example, overexpression of IGFBPs is a technique often used in functional analysis, as is the administration of recombinant protein using *in vitro* cell culture techniques. These methodologies lead to the constitutive presence of the protein, and therefore could result in misleading information being obtained about the functional role of the IGFBPs in growth and development, if, as our data suggests, they have discrete temporal expression patterns. The results described herein highlight the complexities in the regulation of Atlantic salmon IGFBPs.

**Figure 7:**
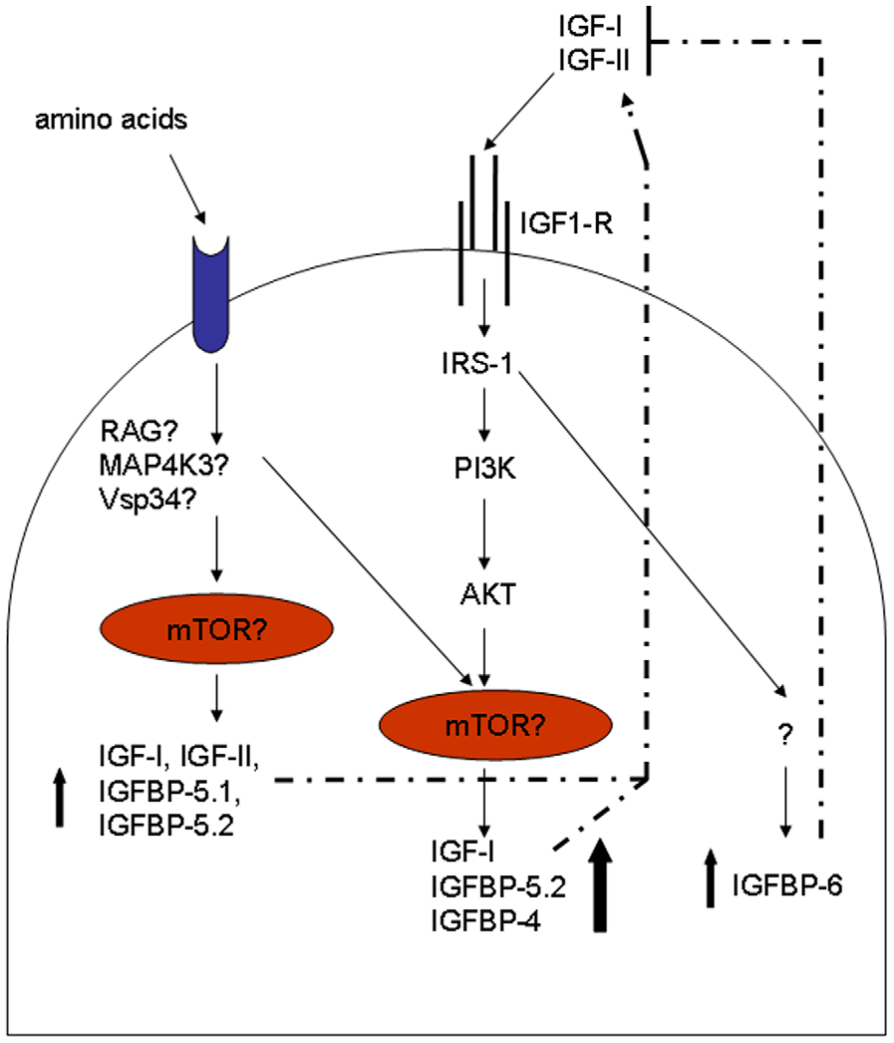
A proposed model of autocrine/paracrine IGF signaling in Atlantic salmon muscle. The model shows amino acid and IGF stimulation of transcription leading to the activation of the myogenic program in Atlantic salmon. It should be noted that we have not measured protein levels, protein activity or protein phosphorylation, so unconfirmed target proteins are indicated by a question mark. Amino acids stimulate *IGFBP-5.1, IGFBP-5.2*, *IGF-I* and *II* expression, which, when translated and secreted from the cell, are able to stimulate the PI3K/AKT/mTOR pathway leading to a positive feedback loop resulting in the synergistically enhanced expression of *IGF-I*, *IGFBP-5.2* and *IGFBP-4*. When amino acids are absent, endocrine and paracrine production of IGF-I and II stimulate *IGFBP-6*, which could serve as a mechanism to sequester available IGFs and thereby inhibit any positive feedback loops.

In conclusion, we have found that amino acids and IGFs separately and together can stimulate the expression of many genes in the IGF signalling pathway. Figure 7 summarises a model of the transcriptional regulation of myogenesis by amino acids and IGFs based on our results. These results highlight the importance of amino acids in regulating myogenesis in Atlantic salmon and demonstrate that amino acids alone are capable of stimulating autocrine/paracrine production of IGFs. The identification of markers specific for amino acid, IGF and synergistic responses will be useful tools for future studies aimed at unravelling the signalling events controlling teleost myogenesis.

## Supporting Information

Figure S1 Correlated gene expression patterns in 3 separate cultures for myoD1b and IGFBP-4 (R = 0.88, P<0.0001, Sup Fig. S1A), IGFBP-4 and myf5 (R = 0.84, P<0.0001 Sup Fig. S1B) and IGF-II and IGFBP-rP1 (R = 0.81, P<0.0001 Sup Fig. S1C).To demonstrate that the correlations are not due to inter-culture variation, the data points for culture 1, 2 and 3 are indicated separately by square, triangle and circle respectively. The combined regression (using data points from all cultures N = 42) is shown.
(0.28 MB TIF)
