# Supplementary figures and images for: Transcriptional Regulation of the IGF Signaling Pathway by Amino Acids and Insulin-Like Growth Factors during Myogenesis in Atlantic Salmon
Source: PLoS One. 2010 Jun 14;5(6):e11100. doi: 10.1371/journal.pone.0011100 (PMC2885424; doi:10.1371/journal.pone.0011100)

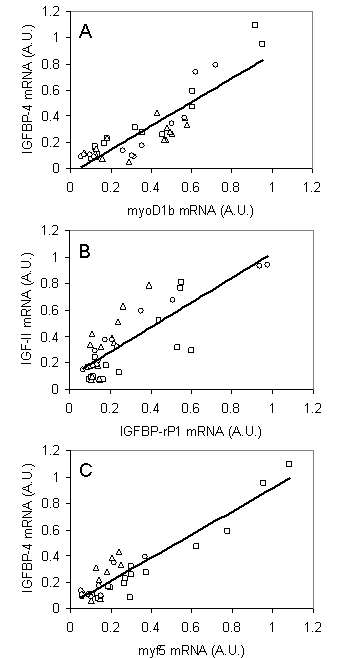

Supplement: Figure S1 — Correlated gene expression patterns in 3 separate cultures for myoD1b and IGFBP-4 (R = 0.88, P<0.0001, Sup Fig. S1A), IGFBP-4 and myf5 (R = 0.84, P<0.0001 Sup Fig. S1B) and IGF-II and IGFBP-rP1 (R = 0.81, P<0.0001 Sup Fig. S1C).To demonstrate that the correlations are not due to inter-culture variation, the data points for culture 1, 2 and 3 are indicated separately by square, triangle and circle respectively. The combined regression (using data points from all cultures N = 42) is shown. (0.28 MB TIF) [file pone.0011100.s001.tif]
